# A Novel Platelet Concentrate: Titanium-Prepared Platelet-Rich Fibrin

**DOI:** 10.1155/2014/209548

**Published:** 2014-01-21

**Authors:** Mustafa Tunalı, Hakan Özdemir, Zafer Küçükodacı, Serhan Akman, Emre Yaprak, Hülya Toker, Erhan Fıratlı

**Affiliations:** ^1^Department of Periodontology, Haydarpasa Training Hospital, Gulhane Military Medical Academy, Üsküdar, 34618 Istanbul, Turkey; ^2^Department of Periodontology, School of Dentistry, Cumhuriyet University, 58140 Sivas, Turkey; ^3^Department of Medical Pathology, Gulhane Military Medical Academy, 34618 Istanbul, Turkey; ^4^Department of Prosthodontics, School of Dentistry, Selcuk University, 42250 Konya, Turkey; ^5^Department of Periodontology, School of Dentistry, Kocaeli University, 41380 Izmit, Turkey; ^6^Department of Periodontology, School of Dentistry, Istanbul University, 34303 Istanbul, Turkey

## Abstract

We developed a new product called titanium-prepared platelet-rich fibrin (T-PRF). The T-PRF method is based on the hypothesis that titanium may be more effective in activating platelets than the silica activators used with glass tubes in Chouckroun's leukocyte- and platelet-rich fibrin (L-PRF) method. In this study, we aimed to define the structural characteristics of T-PRF and compare it with L-PRF. Blood samples were collected from 10 healthy male volunteers. The blood samples were drawn using a syringe. Nine milliliters was transferred to a dry glass tube, and 9 mL was transferred to a titanium tube. Half of each clot (i.e., the blood that was clotted using T-PRF or L-PRF) was processed with a scanning electron microscope (SEM). The other half of each clot was processed for fluorescence microscopy analysis and light microscopy analysis. The T-PRF samples seemed to have a highly organized network with continuous integrity compared to the other L-PRF samples. Histomorphometric analysis showed that T-PRF fibrin network covers larger area than L-PRF fibrin network; also fibrin seemed thicker in the T-PRF samples. This is the first human study to define T-PRF as an autogenous leukocyte- and platelet-rich fibrin product. The platelet activation by titanium seems to offer some high characteristics to T-PRF.

## 1. Introduction

In recent years, there has been a growing interest in the use of platelet-rich products for the treatment of many clinical conditions in dentistry. Platelet-rich fibrin was first developed as an autologous leukocyte- and platelet-rich fibrin (L-PRF) biomaterial in France since 2001 [1]. Unlike other platelet-rich products, this technique requires neither anticoagulant nor bovine thrombin (nor any other gelling agent). Thus, this platelet-rich fibrin is considered a second generation platelet concentrate [2–7]. Without an anticoagulant, most platelets are activated a few minutes after contacting the tube walls, which initiates the coagulation cascade. Fibrinogen is initially concentrated in the upper part of the tube, before the circulating thrombin transforms it into fibrin. A fibrin clot is then formed in the middle of the tube, just between the red corpuscles at the bottom and the acellular plasma at the top [4].

The success of this technique entirely depends on the speed of blood collection and transfer to the centrifuge. Indeed, without the anticoagulant, the blood samples start to coagulate almost immediately upon contact with the tube glass, and it takes only a few minutes of centrifugation to concentrate the fibrinogen in the middle and upper part of the tube [4]. Quick handling is the only way to obtain a clinically usable L-PRF clot. If the time required to collect the blood and launch the centrifugation is overly long, failure will occur. The fibrin will diffusely polymerize in the tube, and only a small blood clot without consistency will be obtained [8].

Successful clinical results have been reported with L-PRF [9–12], but some physicians [13] worry about a possible health hazard with glass-evacuated blood collection tubes with silica activators. O'Connell [13] described the unavoidable silica contact. The silica particles in the tube, although dense enough to sediment with the red blood cells, are small enough for a fraction to remain colloidally suspended in the buffy coat, fibrin, and platelet-poor plasma layers; therefore, these particles might reach the patient when the product is used for treatment.

Although this issue is still debated, the cell composition and three-dimensional organization of L-PRF were evaluated by the influence of different collection tubes (dry glass or glass-coated plastic tubes) and compression procedures (forcible or soft) on the final L-PRF-membrane architecture [14]. It was shown that the type of tested tube (dry glass or glass-coated plastic tubes) and the compression process of the clot (forcible or soft) did not influence the architecture of this second generation platelet concentrate.

Following these discussions, several research groups published studies concerning L-PRF [11, 12, 15–19]; however, none of these studies reported a clinically significant drawback with the glass tubes.

Despite these findings and the successful results in clinical studies, we have modified the initial L-PRF method by changing the structure of the tubes and used a more biocompatible material, titanium [20]. This material was tried to eliminate the speculations about the potential negative effects of silica from dry glass or glass-coated plastic tubes.

In our initial trials, we observed that titanium induced platelet aggregation similar to glass tubes, and the clot produced in titanium tubes was clinically identical to that in glass tubes. In this study, we aimed to define the structural characteristics of this new platelet-rich product prepared in titanium tubes (T-PRF) and compare it with L-PRF.

## 2. Materials and Methods

### 2.1. Preparation of T-PRF and L-PRF Clots

Blood samples were collected from 10 healthy male volunteers (age range from 21 to 35 years) at the GATA Haydarpasa Hospital (Istanbul, Turkey) in February 2011. None of the participants had systemic diseases, and none were smokers. The participants had not taken aspirin or other medications within 2 weeks that could interfere with coagulation. The volunteers provided oral informed consent, and the study was conducted in accordance with the Helsinki Declaration of 1975, as revised in 2000. The study protocol was approved by the Institutional Committee of Ethics in Dental Research of the Faculty of Dentistry at Cumhuriyet University (Sivas, Turkey). The titanium tubes were produced from grade IV titanium. The blood samples were drawn from the antecubital vein of the subject's right or left arm in one attempt (18 mL of blood was drawn by syringe; 9 mL was transferred to the titanium tube and 9 mL was transferred to the dry glass tube). The blood was quickly collected, and the tubes were immediately centrifuged at 2,800 rpm for 12 minutes [4] with a specific table centrifuge, Hettich Universal 320 (Hettich Zentrifugen, Germany), at room temperature. After centrifugation, the T-PRF and L-PRF clots were removed from the tubes using sterile tweezers, separated from the RBC base using scissors, and placed on sterile woven gauze. Each clot (i.e., the blood that was clotted using T-PRF or L-PRF) was left to release its serum. In each series (i.e., the titanium or dry glass tubes), the clots were left on sterile woven gauze to release their serum slowly over 20 minutes. Half of each clot, after sectioning the clot into two parts along its long axis, was processed for SEM evaluation and fixed in 2.5% glutaraldehyde. The other half of each clot was sectioned for a second time into two parts along its long axis; one part was used for the fluorescence microscopy analysis and the other part was used for the light microscopy analysis.

### 2.2. Histological Procedures for Light Microscopy

The T-PRF and L-PRF clots were dehydrated in increasing gradients of alcohol (70%, 95%, and 100%) and placed in toluene before including the paraffin. After complete dehydration, the clot was 0.5 mm thick. For each T-PRF and L-PRF membrane, a series of 20 successive 7-mm sections was created along the long axis of the clot (i.e., 140 mm of the clot thickness could be analyzed in a longitudinal and reliable manner). These 20 sections were stained with hematoxylin and eosin.

### 2.3. Histological Procedures for the Fluorescence Microscopy

The frozen sections of the T-PRF and L-PRF clot specimens were used for the direct immunofluorescent method (DIF) (Fibrinogen, FITC (Ventana, Catalog Number: 760-2685)). An automatic immunohistochemical staining device, Ventana BenchMark XT (Ventana Medical Systems, Inc., Arizona, USA), was used, and the immunofluorescent staining was performed using a human fibrinogen antibody. The sections were evaluated with a fluorescent microscope (Laika DM 2500, Leica and Zeiss Co., Cambridge, England).

### 2.4. Histological Procedures for SEM Evaluation

The morphologic evaluation of the T-PRF and L-PRF clots was conducted with a scanning electron microscope (LEO 440, Leica and Zeiss Co., Cambridge, England). The clots were fixed in 2.5% glutaraldehyde for 1 hour and treated for desiccation. The specimens were sputter coated with 20 nm of gold/palladium and subsequently examined in a scanning electron microscope. Photographs were taken at 20 kV using a magnification from 51 to 5000.

### 2.5. Histomorphometric Analysis

Histometric analysis was performed by examiner blinded with respect to the treatment rendered. Pictures from three different areas of each section (0,348 mm^2^) were taken with an attached camera photofluorescence microscope in groups with an original magnification ×400 (Laika DM 2500, Leica and Zeiss Co., Cambridge, England). The digital images were saved on a computer. The Clemex Vision-Lite 5.0 software (Clemex Technologies, Quebec, Canada) was used for the histomorphometric analysis.

### 2.6. Statistical Analysis

The statistical analysis was performed using a commercially available software programme (SPSS 14.0, SPSS Inc., Chicago, IL, USA). After evaluation of normality with Kolmogorov-Smirnov test, data were analyzed with Mann-Whitney *U* test for pairwise comparisons. *P* values less than 0.05 were considered statistically significant.

## 3. Results

### 3.1. Light Microscopy Study

**Figure 1:**
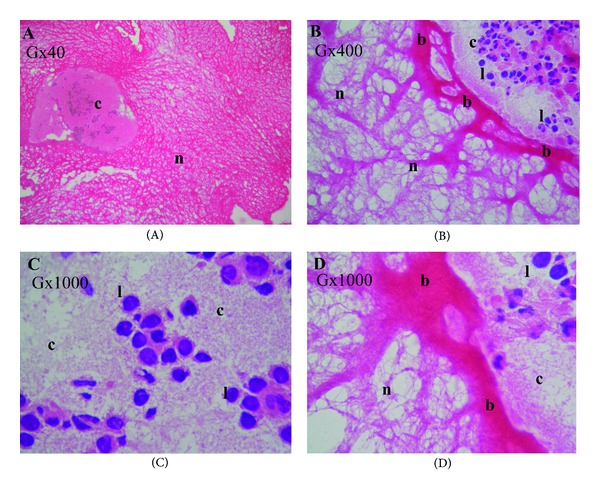
The light microscopy analysis of the T-PRF clot. Hematoxylin and eosin staining. **(A)** The thick T-PRF fibrin network (light pink (n)) and cellular components (c) were not easily detectable. **(B)** The thick border (b) between the cellular structures (c), fibrin network (n), and dark blue leukocyte nuclei (l). **(C) **The cellular components region (c). Leukocytes (l) (dark blue).** (D)** The thick fibrin border (dark pink (b)), cellular structures (c), and well-organized fibrin network with continuous integrity. The magnifications (G) are indicated in each panel.

**Figure 2:**
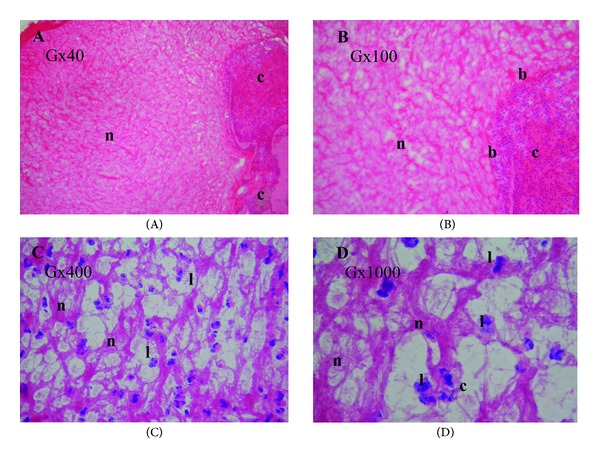
The light microscopy analysis of the L-PRF clot. Hematoxylin and eosin staining. **(A)** The L-PRF fibrin network (light pink (n)) and cellular components (c) were not easily detectable. **(B)** The L-PRF fibrin network (n) adjacent to the cellular area (c) and fibrin border (b) was not easily detectable. **(C)** Dark blue leukocyte nuclei (I), the border between cellular structures and fibrin network (n). **(D)** The cellular structures (c) were embedded in the fibrin network (n). The leukocyte nuclei (l) were stained dark blue with hematoxylin. The magnifications (G) are indicated in each panel.

With the hematoxylin and eosin staining, the fibrin matrix appeared homogeneous in light pink. The RBCs and cytoplasm of the leukocytes were not easily detectable, as they were a darker pink. The leukocyte nuclei were stained dark blue with hematoxylin (Figures 1 and 2). Both T-PRF and L-PRF had a well-organized structure with the hematoxylin and eosin stain under the low-power field (×40 and ×100 magnification) (Figures 1(a), 2(a), and 2(b)). Under a higher magnification of the same samples (×400 and ×1000 magnification), the T-PRF samples showed a highly organized network with continuous integrity compared to the PRF samples (Figures 1(b), 1(c), 1(d)–2(c), and 2(d)). A similar fibrin network structure and cellular components distinguishable from fibrin were observed in both samples (Figures 1(a)–2(a)). The fibrin border between the cellular structures and the fibrin network appeared thicker and more prominent in the T-PRF samples than in the L-PRF samples (Figures 1(b) and 1(d)–2(d)).

### 3.2. Fluorescence Microscopy Study

**Figure 3:**
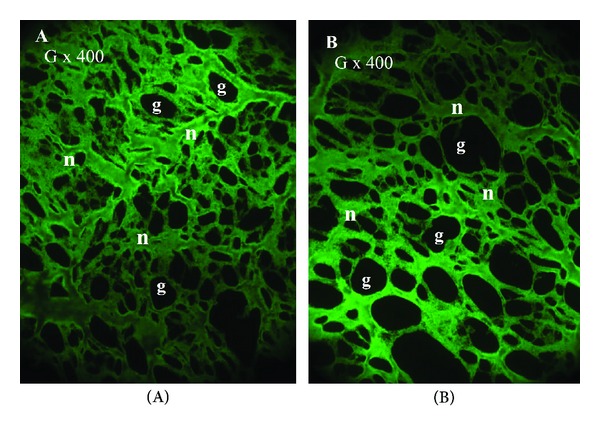
The immunofluorescent microscopy analysis of the T-PRF and L-PRF fibrin network structure. **(A)** The mature and dense T-PRF fibrin network with small gaps (g). **(B)** The mature and dense L-PRF fibrin network with large gaps (g) between the fibrin meshwork. The magnifications (G) are indicated in each panel.

With the immunofluorescent staining, the fibrin network appeared mature and dense in both the T-PRF and L-PRF groups (Figures 3(a) and 3(b)). Although there were slight differences in the architecture between the groups, the main network structure was similar in all samples (Figure 3). The fibrin seemed thicker and better organized in the T-PRF samples (Figures 3(a)-3(b)), but individual differences could have influenced the fibrin structure.

### 3.3. SEM Evaluation

**Figure 4:**
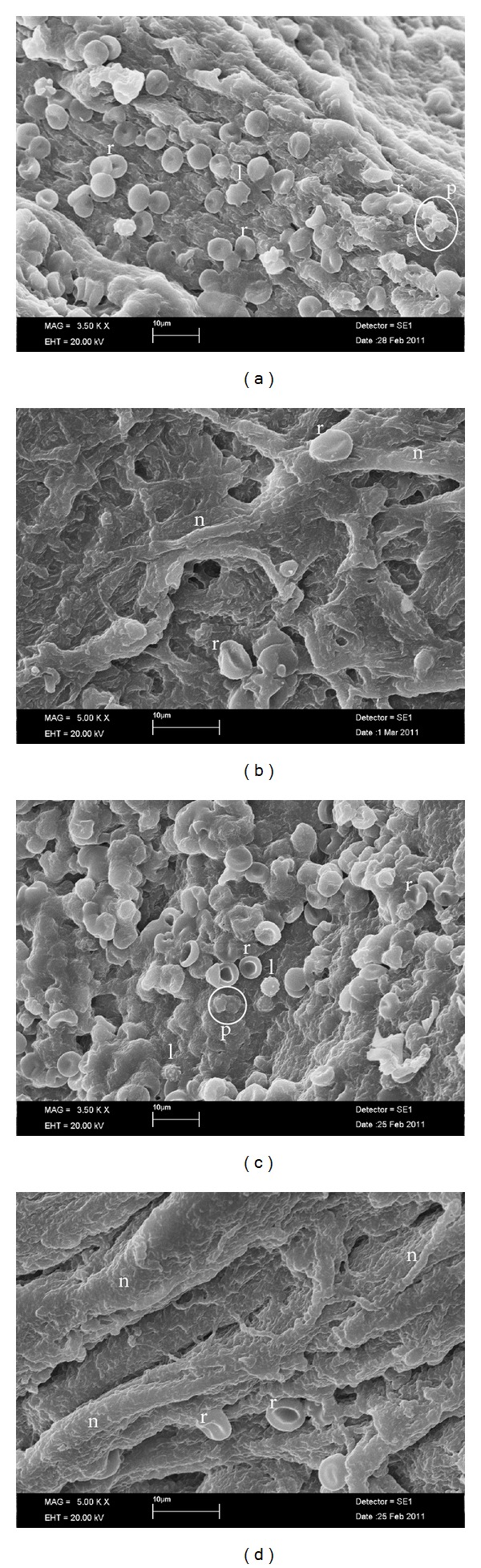
Scanning electron micrograph. (a) The border between the T-PRF fibrin matrix and cellular components. The RBCs (r) trapped within the fibrin matrix. The leukocytes (l) appeared as spherical structures with an irregular surface. The platelets were often enmeshed in the fibrin network but sometimes appeared as aggregates (p (white circle)) (SEM; original magnification ×3500). (b) The T-PRF fibrin matrix. The organized and mature fibrin structure tended to form a network. The RBCs (r) trapped within the fibrin matrix (SEM; original magnification ×5000). (c) The border between the L-PRF fibrin matrix and cellular components. The RBCs (r) were trapped within the fibrin matrix. The leukocytes (l) appeared as spherical structures with an irregular surface. The platelets were often enmeshed in the fibrin network but sometimes appeared as aggregates (p (white circle)) (SEM; original magnification ×3500). (d) The L-PRF fibrin matrix. The organized and mature line-like fibrin structure with thick and thin fibrils. The RBCs (r) were trapped within the fibrin matrix (SEM; original magnification ×5000).

In both groups, the RBCs were located either out of the matrix or adhered to the matrix at the border of the red area and yellow clot. A small number of cellular components and different shaped structures other than RBCs were also observed (Figure 4). A comparison of the images showed a well-organized matrix and fibrin maturation in the T-PRF group. A fen-like matrix was the main picture in both groups. The basic components of the yellow part of the clot were aggregated platelets and fibrin fibrils. RBC-like structures, which are less frequent when compared to the red part, were also noted. It was not possible to determine the shape and type of the structures, other than the platelets embedded in the fibrin matrix. The yellow parts from both groups showed an organized and mature fibrin structure with a tendency to form a network. No other significant difference was detected between these parts of the samples.

At the junction of the red and yellow parts of the T-PRF and L-PRF clots (i.e., the buffy coat area), the SEM examination showed leukocytes that clearly appeared as spherical structures with irregular surfaces (Figure 4). Most seemed quite small (i.e., between 6 and 8 mm in diameter); therefore, these structures could have been lymphocytes.

### 3.4. Histomorphometric Evaluation

**Figure 5:**
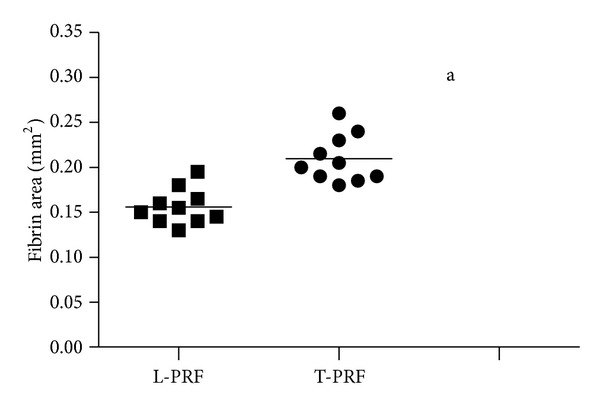
Results of the histomorphometry measurements (mm²). ^a^ *P* < 0.05 versus L-PRF group.

Histometric analysis from fluorescence microscopy images of examples showed that T-PRF fibrin network covers statistically significant larger area than L-PRF fibrin network (Figure 5).

## 4. Discussion

In this study, we developed a new product called T-PRF, which is produced using a modified L-PRF method [1, 4]. To avoid any confusion between the various platelet-rich products and methods to obtain these products, we use Dohan Ehrenfest's classification, which defines four major categories for these products [7]. Platelet-rich products can be classified as follows according to their leukocyte and fibrin content: only platelet-rich plasma products, platelet- and leukocyte-rich plasma products, only platelet-rich fibrin, and platelet- and leukocyte-rich fibrin. According to Dohan Ehrenfest et al. [7], L-PRF method yields a second generation platelet-rich product because no anticoagulant is used. The cell composition of L-PRF implies that this biomaterial is a blood-derived living tissue that must be handled carefully to keep its cellular content alive and stable [21]. Our T-PRF method is based on the hypothesis that it may be potentially beneficial if a titanium tube is used instead of a glass tube in the classical L-PRF method. T-PRF is a platelet- and leukocyte-rich fibrin similar to that obtained from the classical L-PRF method. Although T-PRF and the L-PRF methods are quite similar, the titanium-induced platelet activation provides distinctive characteristics to T-PRF. Titanium has one of the highest strength-to-weight ratios and corrosion resistance among metals [22]. Due to its noncorrosive properties, titanium has excellent biocompatibility [23]. The material passivates itself in vivo by forming an adhesive oxide layer. Titanium also displays a unique property of osseointegration, connecting both structurally and functionally with the underlying bone, and is commonly used in total joint replacements [24], dental implants, internal and external fixators, artificial heart valves, spinal fusion, and medical devices [25]. Hemocompatibility is a key property for biomaterials that come into contact with the blood. Surface modifications have shown great potential for improving the hemocompatibility of biomedical materials and devices [26]. Several authors have discussed the biocompatibility and hemocompatibility of pure titanium [27–30]; therefore, titanium is a promising biocompatible material for biomedical devices, either in the orthopedic or in the cardiovascular field [27].

This first human study of T-PRF in the literature confirmed that the basic histological structure of T-PRF is similar to L-PRF; however, the fibrin of T-PRF seemed more tightly woven and thicker than that of the classic L-PRF (Figure 5). This difference may be due to a better hemocompatibility of titanium compared to glass, which could have potentially led to the formation of a more polymerized fibrin [26]. Due to this structure, we can hypothesize that T-PRF may last a bit longer in the tissue [20]. The SEM examination showed no difference between the two groups in platelet aggregation or the presence of leucocytes.

## 5. Conclusions

In recent years, L-PRF has become increasingly important and has been used in numerous clinical studies [11, 12, 15–19]. Our study will contribute to the improvement of L-PRF and its biocompatibility. This first in vitro study defines T-PRF as an autogenous platelet- and leukocyte-rich fibrin product with a strong fibrin network, but further in vitro and in vivo studies are required to reveal its exact significance. We hope that T-PRF may be widely used in the future, even if the high cost of preparation with titanium tubes, in comparison with glass tubes, may limit the widespread of this new technical option; however, the T-PRF is already an interesting experimental product and more research regarding the clinical parameters of T-PRF, such as the resorption time and its clinical success, is required.
